# DSG2 expression is correlated with poor prognosis and promotes early-stage cervical cancer

**DOI:** 10.1186/s12935-020-01292-x

**Published:** 2020-06-03

**Authors:** Shuhang Qin, Yuandong Liao, Qiqiao Du, Wei Wang, Jiaming Huang, Pan Liu, Chunliang Shang, Tianyu Liu, Meng Xia, Shuzhong Yao

**Affiliations:** 1 Department of Obstetrics and Gynecology, The First Affiliated Hospital, Sun Yat-sen University, Zhongshan Second Road 58, Guangzhou, 510080 People’s Republic of China; 2 Department of Obstetrics and Gynecology, Peking University Third Hospital, Beijing, 100191 People’s Republic of China

**Keywords:** Desmoglein-2, Early-stage cervical cancer, Prognosis, Pelvic lymph node metastasis

## Abstract

**Background:**

The pathogenesis and developmental mechanism of early-stage (FIGO 2009 IA2-IIA2) cervical cancer (CC) remain unclear. Seeking novel molecular biomarkers based on The Cancer Genome Atlas (TCGA) will facilitate the understanding of CC pathogenesis and help evaluate early-stage CC prognosis.

**Methods:**

To identify prognosis-related genes in early-stage CC, we analyzed TCGA mRNA-seq data and clinical data by univariate Cox and Kaplan–Meier plotter analyses. Differential expression analysis identified upregulated genes in early-stage CC. Combined with the genes correlated with unfavorable prognosis, we selected desmoglein-2 (DSG2) for further investigation. To detect DSG2 expression in early-stage CC, we used immunohistochemistry (IHC), quantitative real-time PCR (qRT-PCR) and western blotting. The relationship between the expression of DSG2 and clinical features was analyzed by the Chi square test. Cox analysis was applied to assess the relationship between CC overall survival (OS) and risk factors. The correlations between DSG2 expression and CC cell line proliferation and migration were investigated with Cell Counting Kit-8 (CCK-8) and migration assays.

**Results:**

There were 416 prognosis-related genes in early-stage CC. DSG2, matrix metallopeptidase 1 (MMP1), carbonic anhydrase IX (CA9), homeobox A1 (HOXA1), and serine protease inhibitor B3 (SERPINB3) were upregulated in early-stage CC compared with adjacent noncancerous tissue (ANT) and correlated with unfavorable prognosis. Among them, DSG2 was most significantly correlated with patient survival. Coexpression analysis indicated that DSG2 was probably involved in cell division, positive regulation of transferase activity, positive regulation of cell migration, EGFR upregulation pathway and regulation of lymphangiogenesis. IHC, qRT-PCR and western blotting showed that DSG2 expression was higher in CC than in normal tissue. Significant correlations were identified between DSG2 expression and several aggressive clinical features, including pelvic lymph node metastasis (PLNM). Multivariate Cox analysis showed that DSG2 and PLNM were independent prognostic factors for OS. DSG2 knockdown inhibited CC cell proliferation and migration.

**Conclusions:**

DSG2 is a biomarker that promotes tumor proliferation and metastasis and is correlated with poor prognosis in early-stage CC.

## Background

Uterine cervical cancer (CC) is one of most common cancers. According to the Global Cancer Statistics 2018 report, the incidence rate and mortality of CC ranked fourth among all female cancers [1]. In contrast to late-stage CC patients, most early-stage (IA2-IIA2) CC patients have a significantly increased survival time after surgery and chemoradiotherapy. However, approximately 10–30% of early-stage patients were found to have pelvic lymph node metastasis (PLNM), and some of the patients eventually experienced adverse outcomes [2]. In early-stage CC, patients with moderately high-risk factors, including large tumor size (> 2 cm), poor differentiation, special pathologic types, deep stromal invasion, lymphovascular space invasion (LVSI), PLNM and parametrial infiltration, usually have relatively shorter survival times [2, 3].

Currently, the pathogenesis and mechanism of CC metastasis remain unclear and probably involve the aberrant expression of numerous oncogenes and tumor suppressors. Rapid advances in molecular biotechnology revealed that some molecular biomarkers are related to the progression of CC [4]. Seeking novel molecular biomarkers of protein-coding genes would facilitate the understanding of CC pathogenesis and help us evaluate the prognosis of early-stage CC.

The Cancer Genome Atlas (TCGA) database has been developed in recent years. It is composed of a large amount of cancer mRNA-seq data as well as detailed clinical data, which makes bioinformatic data mining convenient and reliable [5]. We incorporated gene profiling, molecular signatures, and functional and pathway information with gene set enrichment analysis. Using bioinformatics analyses, we found a series of early-stage CC prognosis-related genes. Among all these genes, we found that desmoglein-2 (DSG2) was upregulated in early CC compared with normal samples and also predicted unfavorable prognosis in early CC.

DSG2 is a cell adhesion protein of the cadherin superfamily that is crucial for cardiomyocyte cohesion and function [6]. Its purpose is to regulate cell–cell contact with adjacent cells. The altered expression and function of desmosomal cadherins is associated with human tumorigenesis [7]. Brennan et al. [8] and Kurzen et al. [9] showed that DSG2 was more highly expressed in skin squamous cell carcinoma and basal cell carcinoma and that the positive rate was higher in high-risk patients. Kamekura et al. [10] showed that the downregulation of DSG2 inhibited the proliferation of colon cancer cells. Saaber et al. [11] showed that DSG2 was a novel biomarker of squamous cell lung carcinoma. Cai et al. [12] showed that DSG2 was more highly expressed in non-small cell lung cancer (NSCLC) and that the knockdown of DSG2 inhibited the progression of NSCLC. However, some studies have shown that DSG2 is expressed at lower levels in cancer and functions as a tumor suppressor. Yashiro et al. [13] showed that the high expression of DSG2 was correlated with a longer survival time among diffuse infiltrative carcinomas of the stomach. Ramani et al. [14] showed that the knockdown of DSG2 decreased the cell junction of pancreatic carcinoma cells and increased the rate of metastasis. Barber et al. [15] showed that the low expression of DSG2 was an independent prognostic factor for prostate cancer. Davies et al. [16] showed that lower-expressed DSG2 was correlated with poor differentiation, larger tumor size and lymph node metastasis in breast cancer.

However, the role of DSG2 in CC has never been explored. In the present study, we identified DSG2 as a novel CC prognosis-related gene using data mining. With clinical and cell line validation, we demonstrated that it probably increased the risk of PLNM and resulted in an unfavorable prognosis.

## Methods

### Datasets

The gene expression and clinicopathological data of 310 CC patients and 3 adjacent noncancerous tissues (ANTs) were downloaded from TCGA (https://portal.gdc.cancer.gov/) [17, 18]. According to the TCGA publication guidelines (https://www.cancer.gov/about-nci/organization/ccg/research/structural-genomics/tcga), these mRNA sequencing data have no restrictions on publication, and no additional approval by an ethics committee was required to publish the use of the data.

With the Ensembl platform (http://www.ensembl.org/), we separated the mRNAs from all the TCGA genes. Genes that had missing values in over 50% of the samples were removed. Finally, there were 12,084 genes included in the study. Samples without data on the survival state and survival time were also removed. Finally, 291 CC tissues, including 167 early-stage (FIGO 2009 IA2-IIA2) CC tissues and 3 ANTs, were included in the study. For the early-stage samples, any missing data on whether LVSI and corpus involvement occurred were all recorded as nonoccurrence (median of the available data).

Four CC datasets from Oncomine (version 4.5) (https://www.oncomine.org/) [19] were used to validate the results obtained from TCGA.

### Kaplan–Meier (KM), univariate Cox, Gene Ontology (GO), Kyoto Encyclopedia of Genes and Genomes (KEGG) and protein–protein interaction (PPI) analyses

The prognostic value of each gene was calculated in the KM analyses and univariate Cox analyses for the early-stage cohort. A total of 416 genes with both *P*_*KM*_ < 0.05 and *P*_*Cox*_< 0.05 were early-stage prognosis-related genes and were kept for further analyses. GO biological process, cellular component, and molecular function categories and KEGG pathway analyses and PPI network construction were conducted by the Metascape website (http://metascape.org/gp/index.html), using false discovery rate (FDR) q-value < 0.05 as the standard for statistical significance.

### Differential expression analyses (DEA)

To identify genes that are more highly expressed in early-stage CC than in ANTs, we performed a DEA of prognosis-related genes between 167 early-stage CC patients and 3 ANTs with the R package “DEseq 2”. The differentially expressed mRNAs with log_2_|FC| > 1.5 and *P*-*adjusted* < 0.05 were considered to be significant. Hierarchical clustering analysis was applied to categorize the data into two groups with similar expression patterns between early-stage CC and ANTs.

### Coexpression analyses

Coexpression analyses was conducted by the cBioPortal website (https://www.cbioportal.org/). Using Spearman’s correlation analyses, the genes with FDR q-value < 0.05 were regarded as coexpressed with DSG2. Then, GO biological process analysis and oncogenic signature analysis were conducted among the positively correlated genes (Spearman’s correlation > 0) and negatively correlated genes (Spearman’s correlation < 0) by the Metascape website.

### Tissue sample collection

A total of 150 CC tissues, 6 ANTs and 30 normal cervical tissues (NCTs) collected from January 2006 to October 2012 were obtained from the archives of the Pathology Department and Gynecology Department of the First Affiliated Hospital of Sun Yat-sen University. All enrolled CC patients were matched from stage IA2 to IIA2 and underwent radical hysterectomy and lymphadenectomy. Only patients with no preoperative radiotherapy or chemotherapy and with available clinical follow-up data were enrolled. Thirty NCTs were collected from patients who underwent hysterectomy without malignant conditions. Written informed consent was obtained from each patient. All specimens were handled according to legal and ethical standards.

### Cell lines and cell culture

In this study, SiHa, HeLa, C33A, CaSki, MS751 and ME180 cells were purchased from the American Type Culture Collection (ATCC, Rockville, MD, USA) and cultured according to their guidelines in a humidified atmosphere with 5% CO_2_ at 37 °C. The SiHa, HeLa and ME180 cell lines were cultured in DMEM (Thermo Fisher, America). The CaSki cell line was cultured in RPMI 1640 medium (Thermo Fisher, America). The C33A and MS751 cell lines were cultured in Eagle’s minimum essential medium (Thermo Fisher, America). The media were supplemented with 10% fetal bovine serum (Life Technology, America) and 1% antibiotics (100 U/ml penicillin and 100 µg/ml streptomycin) (Life Technology, America).

### Immunohistochemistry (IHC)

For IHC, 4-µm paraffin-embedded sections were baked at 60 °C for 1 h, deparaffinized with xylene, rehydrated with a series of graded alcohols, and microwaved in EDTA antigen retrieval buffer. Then, the sections were blocked with 10% goat serum before incubation with a primary antibody at 4 °C overnight, followed by HRP-conjugated secondary antibody incubation for 30 min at room temperature. DAB was added to detect antibody binding. Once brown color appeared, the sections were immersed in distilled water to stop the reaction. The sections were counterstained with hematoxylin, dehydrated in graded alcohols and mounted. The primary antibodies were rabbit anti-human DSG2 monoclonal antibody (ab150372, Abcam, Britain) and mouse anti-human D2–40 monoclonal antibody (MAB-0567, MXB, China). The DSG2 staining results were scored based on the following criteria: (i) percentage of positive tumor cells in the tumor tissue: 0 (0%), 1 (1–10%), 2 (11–50%), 3 (51–70%) and 4 (71–100%); and (ii) staining intensity: 0 (none), 1 (weak), 2 (moderate), and 3 (strong). The staining index was calculated as the staining intensity score × the proportion of positive tumor cells (range from 0 to 12). The staining score of 6 was defined as the cutoff. Thus, patients with different positive staining levels of DSG2 expression were divided into low- and high-staining groups.

### RNA extraction and quantitative real-time PCR (qRT-PCR)

Total RNA was extracted using Trizol reagent (TAKARA, Japan) according to the manufacturer’s instructions, and the concentration of the RNA extracts of each sample was measured quantitatively by a NanoDrop ND-2000 spectrophotometer. RNA was reverse transcribed into cDNA by using PrimeScript RT Master Mix (TAKARA, Japan). cDNA was amplified and quantified using a 7500 Fast Real-Time PCR system (Applied Biosystems, USA) and SYBR Premix Ex Taq (TAKARA, Japan). The RT–PCR conditions for genes were set at 95 °C for 2 min, followed by 39 cycles at 95 °C for 20 s, 58 °C for 30 s and 72 °C for 30 s. The DSG2 sequences were 5′-CTCAGGTGTGCAGCCTACTC-3′ (forward) and 5′-GTGGTGTTCCTAGCCGTCAT-3′ (reverse), while the GAPDH sequences were 5′-TGCACCACCAACTGCTTAGC-3′ (forward) and 5′-GGCATGGACTGTGGTCATGAG-3′ (reverse). qRT-PCR was repeated at least three times. mRNA expression was defined based on Ct, and relative expression levels were calculated using the comparative Ct (2^−ΔΔCt^) method after normalization with reference to the expression of the house-keeping gene GAPDH.

### Western blot assay

Total protein was extracted with cold RIPA lysis buffer and fractionated by sodium dodecyl sulfate–polyacrylamide gel electrophoresis (SDS-PAGE) and then transferred onto a 0.45-μm PVDF membrane (Millipore, America). The membranes were blocked with 5% skimmed milk and incubated with the primary antibody at 4 °C overnight, followed by secondary antibody incubation for 1 h at room temperature. Bound antibodies were detected with Immobilon Western Chemiluminescent HRP Substrate (Millipore, America). Rabbit anti-human DSG2 monoclonal antibody (ab150372, Abcam, Britain) and rabbit anti-human GAPDH antibody (XS20180808002, Bioworld, China) were used in this study.

### siRNA-mediated knockdown of DSG2

SiHa and HeLa cells were transfected with control siRNA (GenePharma, Shanghai, China) or DSG2-specific siRNA (GenePharma, China) using Lipofectamine RNAiMAX Reagent (Invitrogen, America) and Opti-MEM media (Life Technology, America) at the time of cell culture. There were two DSG2 siRNA sequences. The siRNA393 sequences were 5′-CCAAUUGCCAAGAUACAUUTT-3′ (forward) and 5′-AAUGUAUCUUGGCAAUUGGTT-3′ (reverse). The siRNA613 sequences were 5′-CCUUAGAGCUACGCAUUAATT-3′ (forward) and 5′-UUAAUGCGUAGCUCUAAGGTT-3′ (reverse). The negative control sequence (siRNA-NC) was 5′-UUCUCCGAACGUGUCACGUTT-3′ (forward) and 5′-ACGUGACACGUUCGGAGAATT-3′ (reverse).

### Cell Counting Kit-8 (CCK-8) assay

For the CCK-8 assay, 5 × 10^3^ SiHa and HeLa cells were seeded into each well of 96-well plates. The time calculation started when the cells adhered to the wall, and the wells were transfected with siRNA. Cell viability was measured at specific times by CCK-8 (CCK-8, DOJINDO, Japan). The absorbance value at 450 nm was read by a microplate reader (Tecan Sunrise, Tecan Group Ltd.).

### Migration assay

The stable cell lines SiHa siRNA-NC, SiHa siRNA393, SiHa siRNA613, HeLa siRNA-NC, HeLa siRNA393 and HeLa siRNA613 were counted and then 10 × 10^4^ stably infected SiHa cells and 20 × 10^4^ stably infected HeLa cells in 250 µl of serum-free medium were separately plated into the upper chamber of 8-µm transwell inserts (BD Biosciences, Franklin Lakes, NJ), while 500 µl of medium containing 10% bovine serum albumin was added to the lower chamber. After 24 h of incubation at 37 °C, SiHa siRNA cells in the upper chamber were removed carefully. After 48 h of incubation at 37 °C, HeLa siRNA-NC and HeLa siRNA cells in the upper chamber were removed. Migrated cells on the lower membrane surface were fixed in 4% paraformaldehyde (Solarbio, Beijing, China) for 10 min and then stained with 0.1% crystal violet (KeyGEN biotech, Nanjing, China) for 10 min. The number of cells was counted in 5 randomly selected visual fields (100×) per well under an inverted microscope DMI4000B (Leica, Wetzlar, Germany).

### Statistical analyses

Statistical analyses were performed using SPSS 22.0 statistical software (Chicago, IL, USA) and R version 3.6.0. The differences between two groups were analyzed by Student’s t test. The differences among more than two groups were analyzed by ANOVA. The Chi square test and Fisher’s exact test were used to analyze the relationship between DSG2 expression and the clinicopathological characteristics. Survival data were evaluated using univariate and multivariate Cox regression analyses. Survival curves were plotted by the KM method and compared using the log-rank test. In all cases, *P *< 0.05 was considered statistically significant.

## Results

### Early-stage CC prognosis-related genes were identified by bioinformatic analyses

**Fig. 1:**
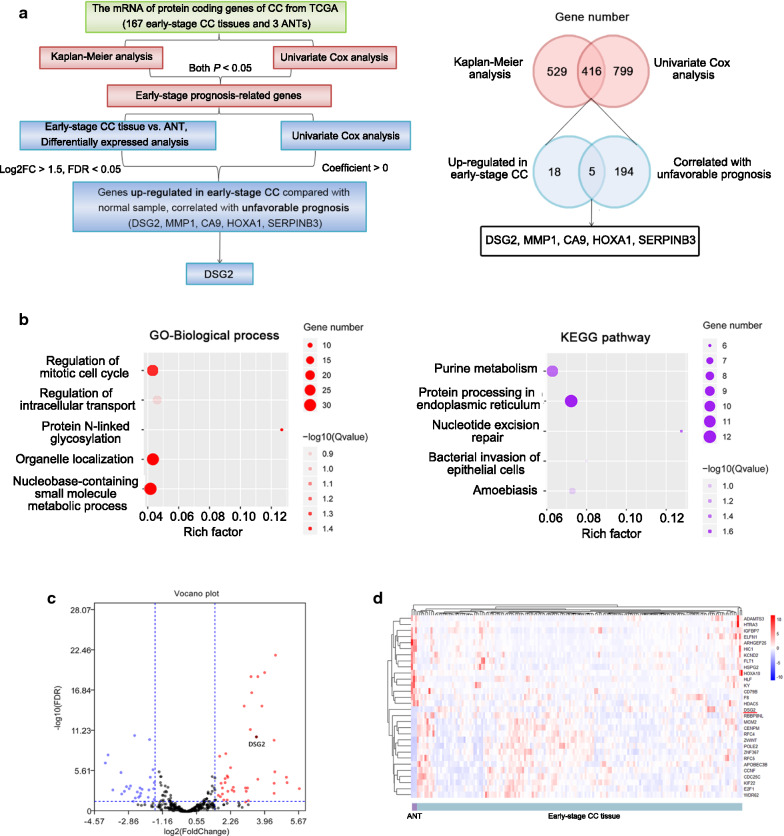
The identification of DSG2 by bioinformatics. **a** Workflow for screening DSG2. **b** GO terms identified in the GO analysis for correlated coding genes in the biological process categories with 5 minimum *P-adjusted* values. Biological pathways from KEGG analysis with 5 minimum *P-adjusted* values. Each dot represents a specific term, with the count number and the corresponding *P-adjusted* value indicated by the size and the color of the dot, respectively. **c** Volcano plot of differentially expressed genes (DEGs) between early-stage CC tissue and adjacent noncancerous tissue (ANT). The genes included in the analysis are prognosis-related genes. **d** Heatmap of the top 15 up- and downregulated DEGs according to the false discover rate (FDR). Red represents high expression, and blue represents low expression

According to the KM plotter analyses and univariate Cox analyses, the TCGA data included 416 early-stage prognosis-related genes, including 217 protective (Cox coefficient < 0) and 199 hazardous (Cox coefficient > 0) genes. In the GO analyses using all survival-related genes, 4 biological process terms were significantly enriched (*P*-*adjusted* < 0.05), including the regulation of the mitotic cell cycle, the nucleobase-containing small molecule metabolic process, protein N-linked glycosylation, and organelle localization. GO cellular component analyses identified endoplasmic reticulum lumen as the significantly enriched signature (*P*-*adjusted* < 0.05). No signatures were significantly enriched in GO molecular function analysis. KEGG pathway analyses indicated that the significant pathways were purine metabolism, protein processing in the endoplasmic reticulum, and nucleotide excision repair (Fig. 1b, Additional file 1: Figure S1a). Each chromosome had different numbers of up- and downregulated prognosis-related genes (Additional file 1: Figure S1b). Additionally, we constructed a PPI network to interpret the potential biological roles of the prognosis-related mRNAs in early-stage CC (Additional file 1: Figure S1c).

### DSG2 was identified by bioinformatic analyses

DEA of prognosis-related genes identified 24 upregulated genes and 171 downregulated genes in CC compared with ANTs. The volcano plot and heatmap of the differentially expressed genes (DEGs) are shown in Fig. 1c, d. DSG2 was included in the top 15 upregulated genes. Five overlapping genes between hazardous (Cox coefficient > 0, *P*_*KM*_ < 0.05 and *P*_*Cox*_ < 0.05) genes and upregulated genes (Log_2_FC > 1.5, FDR < 0.05) in CC were identified. Except for DSG2, the other four genes have been explored in CC. Therefore, DSG2 was used for further validation in clinical samples and cells. The workflow for screening DSG2 and the 5 overlapping genes were shown in Fig. 1a and Table 1.

**Table 1:** The up-regulated genes in early-stage CC tissue compared with ANT, correlated with unfavorable prognosis

Gene	Description
DSG2	Desmoglein-2
MMP1	Matrix metallopeptidase 1
CA9	Carbonic anhydrase IX
HOXA1	Homeobox A1
SERPINB3	Serine protease inhibitor B3

### The potential functions of DSG2 in CC and other cancers were analyzed by bioinformatics

**Fig. 2:**
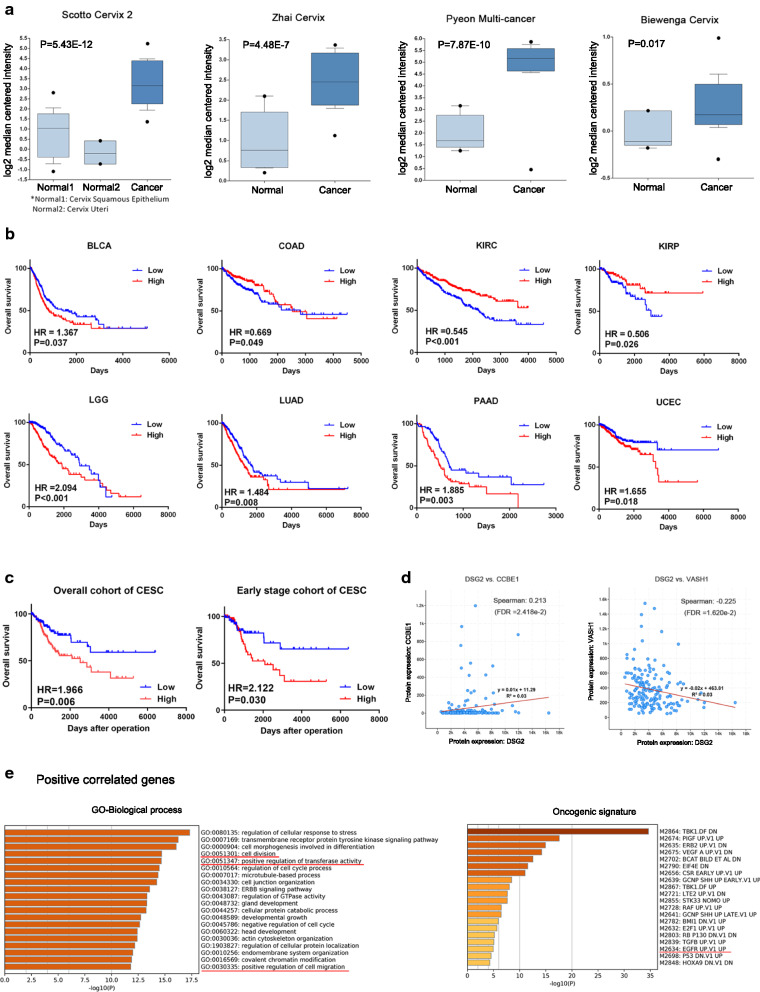
The potential functions of DSG2 in CC and other cancers. **a** Box plots of DSG2 expression based on Oncomine datasets. DSG2 was significantly overexpressed in cancer in the Scotto Cervix 2, Zhai Cervix, Pyeon Multi-cancer and Biewenga Cervix datasets. **b** Kaplan–Meier survival curves of DSG2 showing the overall survival outcomes by relatively high DSG2 expression and low DSG2 expression in patients with cancers except CC. **c** Kaplan–Meier survival curves of DSG2 showing the overall survival outcomes in the overall cohort and early-stage cohort by relatively high DSG2 expression and low DSG2 expression in CC patients. **d** The correlation between DSG2 and two genes regulating lymphangiogenesis. **e** GO terms identified in the GO biological process analysis for positively coexpressed genes in categories with 20 minimum * P-adjusted* values. Oncogenic signature for positively coexpressed genes with 20 minimum *P-adjusted* values

For further validation, we investigated the difference in DSG2 expression between normal tissue and CC based on Oncomine datasets (Fig. 2a). All datasets revealed that DSG2 was upregulated in the cancer group. Survival analyses of both the overall cohort and early-stage cohort showed that the expression of DSG2 predicted an unfavorable prognosis in CC (overall cohort: HR = 1.966, *P *= 0.006; early-stage cohort: HR = 2.122, *P *= 0.030) (Fig. 2c). Furthermore, the overall cohort survival analyses showed that the expression of DSG2 predicted an unfavorable prognosis in bladder urothelial carcinoma (BLCA), brain lower-grade glioma (LGG), lung adenocarcinoma (LUAD), pancreatic adenocarcinoma (PAAD) and uterine corpus endometrial carcinoma (UCEC), while predicting a favorable prognosis in colon adenocarcinoma (COAD), kidney renal clear cell carcinoma (KIRC) and kidney renal papillary cell carcinoma (KIRP) (all *P *< 0.05) (Fig. 2b).

Genes that were coexpressed in conjunction with DSG2 were identified with cBioPortal analyses (*P*-*adjusted* < 0.05). There were 2610 positively correlated genes (Spearman’s correlation > 0) and 2737 negatively correlated genes (Spearman’s correlation < 0). The enriched GO biological process and oncogenic pathway items are shown in Fig. 2e and Additional file 1: Figure S1d. According to the positively correlated gene enrichment, cell division, positive regulation of transferase activity, positive regulation of cell migration and the EGFR upregulation pathway were significantly enriched (*P*-*adjusted* < 0.05), revealing that DSG2 is involved in the process and metastasis of CC. Furthermore, two genes were significantly coexpressed with DSG2, CCBE1 and VASH1, which are genes that regulate lymphangiogenesis according to GO biological process analyses (Fig. 2d). DSG2 was positively correlated with CCBE1, which positively regulated lymphangiogenesis, while it was negatively correlated with VASH1, which negatively regulated lymphangiogenesis.

These results confirmed that DSG2 was important in the development of various cancers and was possibly an oncogenic gene in CC.

### DSG2 expression is upregulated in CC tissues

**Fig. 3:**
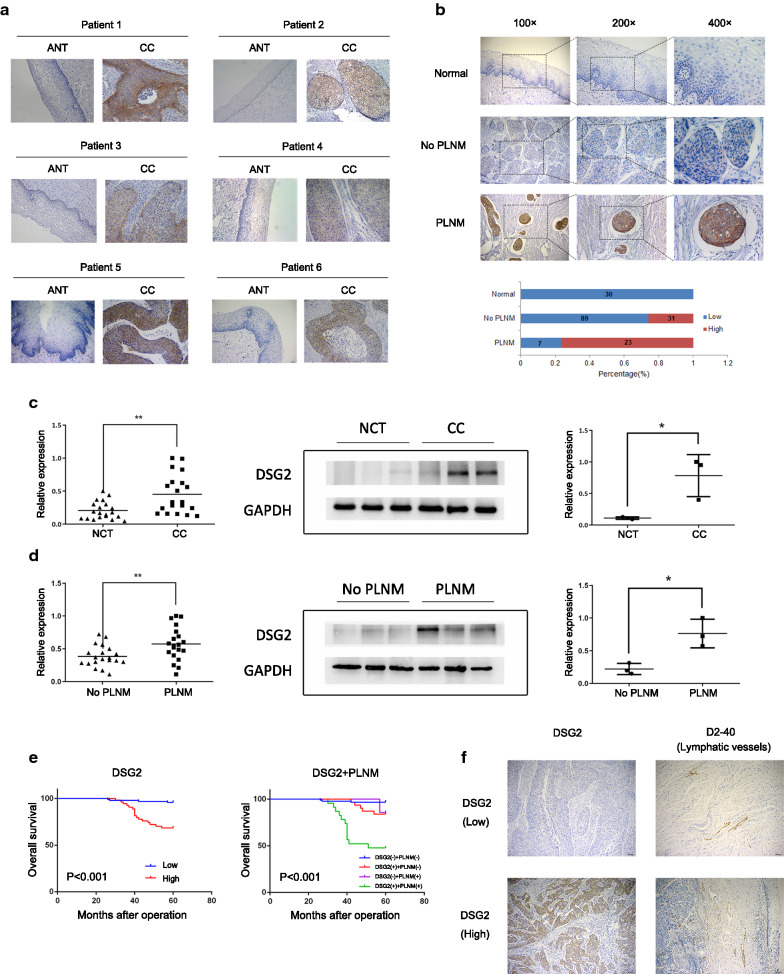
The expression of DSG2 in patient samples was determined by IHC, qRT-PCR and western blotting. **a** IHC was performed on six pairs of matched CC samples and adjacent nontumor cervical tissue (ANT) samples. Original magnifications: ×200. **b** Representative results of the IHC of DSG2 in normal samples and early-stage CC samples with and without PLNM. Bar graph shows the percentage of high/low expression of DSG2 in normal samples and early-stage cohort samples. **c** The mRNA expression of DSG2 was determined by qRT-PCR in 20 NCTs and 20 CC samples (left). The protein expression of DSG2 was determined by western blotting in 3 NCTs and 3 CC samples (right). **d** The mRNA expression of DSG2 was determined by qRT-PCR in 20 CC samples without PLNM and 20 cervical cancer samples with PLNM (left). The protein expression of DSG2 was determined by western blotting in 3 CC samples without PLNM and 3 CC samples with PLNM (right). **e** Kaplan–Meier curve for DSG2 and PLNM in early-stage cohort CC patients. DSG2 (−): low expression of DSG2; DSG2 (+): high expression of DSG2. PLNM (−): without PLNM; PLNM (+): with PLNM. **f** Representative results of the IHC of lymphatic vessel density in CC with high/low expression of DSG2. Original magnifications: 100×

IHC was performed on 150 early-stage CC samples and 30 NCTs, and the results revealed that DSG2 was more highly expressed in CC samples (Fig. 3b). Furthermore, DSG2 expression was upregulated in six early-stage CC samples compared to that in matched ANTs derived from the same patients (Fig. 3a).

Additionally, to validate the results above, the DSG2 mRNA expression level was determined in 20 NCTs and 20 early-stage CC tissues using qRT-PCR. Moreover, 3 NCT and 3 early-stage CC tissues were randomly selected from the abovementioned tissues for western blot analyses. A comparison of the results showed that DSG2 mRNA and protein levels were higher in early-stage CC tissues than in NCTs (Fig. 3c).

### High expression of DSG2 is associated with poor clinical features and prognosis in early-stage CC

The correlation between DSG2 expression and clinicopathological features was analyzed according to the IHC score. High DSG2 expression was significantly correlated with several poor clinicopathological features, including tumor size (*P* = 0.022), PLNM (*P* < 0.001), recurrence (*P* < 0.001) and vital status in 5 years (*P* < 0.001) (Table 2). No significant correlation was identified between DSG2 expression and age, FIGO stage, pathologic type, differentiation grade, stromal invasion, LVSI, vaginal involvement and parametrial infiltration (Table 2). To verify the relationship between DSG2 expression and the prognosis of early-stage CC, univariate and multivariate Cox analyses were performed. Univariate analysis showed that DSG2 expression (*P* < 0.001), tumor size (*P* = 0.029), LVSI (*P* = 0.008) and PLNM (*P* < 0.001) were prognostic factors for overall survival (OS) (Table 3). Multivariate analysis showed that DSG2 expression (*P* = 0.018) and PLNM (*P* = 0.006) were independent prognostic factors for OS (Table 4, Fig. 3e).

**Table 2:** Correlation between DSG2 expression and clinicopathological features of early-stage CC

Variables	Total	DSG2 expression		*P*^a^
		Low	High	
Age (years)				
≤ 42	65	42	23	0.313
> 42	85	48	37	
FIGO stage^b^				
I	132	87	45	0.187
II	18	9	9	
Tumor size (cm)				
≤ 4	125	85	40	*0.022*
> 4	25	11	14	
Pathologic types				
Squamous cell carcinoma	128	82	46	0.205^a^
Adenocarcinoma	18	13	5	
Adenosquamous carcinoma	4	1	3	
Differentiation grade				
Well	5	4	1	0.502^a^
Moderate	69	45	24	
Poor	69	43	26	
Stromal invasion				
< 1/2	87	58	29	0.424
≥ 1/2	63	38	25	
Lymphovascular space invasion				
No	134	89	45	0.074
Yes	16	7	9	
Pelvic lymph node metastasis				
No	120	89	31	< *0.001*
Yes	30	7	23	
Vaginal involvement				
No	148	94	54	0.408
Yes	2	2	0	
Parametrial infiltration				
No	149	95	54	0.64
Yes	1	1	0	
Recurrence				
No	126	89	37	< *0.001*
Yes	24	7	17	
Vital status in 5 years				
Alive	129	92	37	< *0.001*
Dead	21	4	17	

^a^*P* value from Fisher’s exact test; The italic number inside the table reflected *P* < 0.05

^b^FIGO 2009 was used

**Table 3:** Univariate Cox analysis of factors associated with overall survival in early-stage cohort

Variables	HR (95% CI)^a^	*P*
Age (years)	1.007 (0.960–1.056)	0.774
Tumor size (cm)		
≤ 4 (reference)	1	
> 4	2.750 (1.110–6.814)	*0.029*
FIGO stage^b^		
I (reference)	1	
II	0.812 (0.239–2.757)	0.739
Pathologic types		
Squamous cell carcinoma (reference)	1	
Adenocarcinoma	2.605 (0.946–7.171)	0.064
Adenosquamous carcinoma	2.583 (0.341–19.558)	0.358
Differentiation grade		
Well (reference)	1	
Moderate	6009.923 (–)	0.94
Poor	12,458.308 (–)	0.935
Stromal invasion		
<1/2 (reference)	1	
≥1/2	0.681 (0.275–1.687)	0.406
Lymphovascular space invasion		
No (reference)	1	
Yes	3.622 (1.403–9.349)	*0.008*
Pelvic lymph node metastasis		
No (reference)	1	
Yes	8.297 (3.428–20.078)	< *0.001*
Vaginal involvement		
No (reference)	1	
Yes	0.049 (0–483,831.748)	0.713
Parametrial infiltration		
No (reference)	1	
Yes	0.049 (0–368,184,919.781)	0.795
DSG2		
Low (reference)	1	
High	8.679 (2.917–25.822)	< *0.001*

^a^95% CI, 95% confidence interval; HR, hazard ratio

^b^FIGO 2009 was used

**Table 4:** Multivariate Cox analysis of factors associated with overall survival in early-stage cohort

Variables	HR (95% CI)^a^	*P*
Tumor size (cm)		
≤ 4 (reference)	1	
> 4	1.704 (0.646–4.498)	0.282
Lymphovascular space invasion		
No (reference)	1	
Yes	2.595 (0.946–7.123)	0.064
Pelvic lymph node metastasis		
No (reference)	1	
Yes	3.935 (1.480–10.465)	*0.006*
DSG2		
Low (reference)	1	
High	4.234 (1.275–14.063)	*0.018*

^a^ 95% CI, 95% confidence interval; HR, hazard ratio

### High DSG2 expression was correlated with the occurrence of PLNM

The IHC results showed that DSG2 expression was significantly correlated with PLNM (Table 2, Fig. 3b). For further validation, qRT-PCR was performed to examine the mRNA levels of DSG2 in 20 PLNM and 20 non-PLNM tissues, while western blotting was performed to examine the protein levels of DSG2 in 3 PLNM and 3 non-PLNM tissues. Both the mRNA and protein levels of DSG2 in the PLNM group were higher than those in the non-PLNM group (*P *< 0.05) (Fig. 3d). This validation result was consistent with the IHC result.

Moreover, to explore the mechanism of how DSG2 promoted PLNM, we detected the lymphatic microvessel density (LMVD) in the same IHC samples. We found that the high DSG2 expression group had higher LMVD than the low DSG2 group, indicating that DSG2 probably promoted PLNM by promoting lymphangiogenesis (Table 5, Fig. 3f).

**Table 5:** Correlation between DSG2 expression and lymphatic vessel density of early-stage CC

	DSG2 expression		*P*
	Low	High	
Lymphatic vessel density	6.2 ± 3.4	13.4 ± 2.8	*0.034*

### Knockdown of DSG2 expression decreased CC cell proliferation and migration

**Fig. 4:**
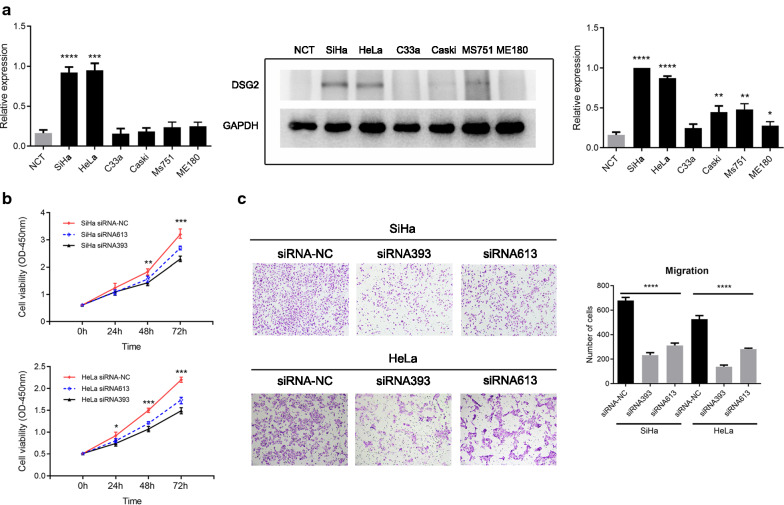
The effect of DSG2 knockdown on the viability and migration of CC cells. **a** Analysis of the expression of DSG2 in six CC cell lines and NCTs by qRT-PCR (left) and western blotting (right). **b** The effect of siRNA on the viability of CC cells detected by CCK-8 assays. **c** The effect of siRNA on the migration abilities of CC cells detected by migration assay. Original magnification: ×100. *P < 0.05; **P < 0.01; ***P < 0.001; ****P < 0.0001

To determine the function of DSG2 in CC cell proliferation and migration, further investigation was performed using the CCK-8 assay and migration assay. First, qRT-PCR and western blotting revealed that DSG2 expression in SiHa and HeLa cells was higher than that in other cells and NCT (Fig. 4a). Therefore, SiHa and HeLa were chosen for further experiments. DSG2 expression was downregulated in SiHa and HeLa cell lines by transfection of siRNA393 and siRNA613. The efficiencies of interference were confirmed by qRT-PCR and western blotting (Additional file 1: Figure S2). The CCK-8 assay showed that knockdown of DSG2 expression decreased the cell proliferative capacity (Fig. 4b). Migration assays showed that knockdown of DSG2 expression decreased cell migration (Fig. 4c).

## Discussion

Using survival analyses and TCGA data, our study provided a series of prognosis-related genes of early-stage CC. With GO, KEGG and PPI analyses, we can determine the main function distribution and interaction of genes. We identified 5 genes that were upregulated in early CC compared with normal samples that were correlated with unfavorable prognosis, including DSG2, matrix metallopeptidase 1 (MMP1), carbonic anhydrase IX (CA9), homeobox A1 (HOXA1), and serine protease inhibitor B3 (SERPINB3). MMP1, CA9, HOXA1 and SERPINB3 had been explored. However, we could not find any studies exploring the relationship between DSG2 and CC. To the best of our knowledge, this is the first one.

Consistent with the TCGA data mining result, DSG2 was more highly expressed in CC tissue than in normal tissue in 4 Oncomine databases. By detecting DSG2 expression in tissues, we revealed that DSG2 was upregulated in CC tissue compared with ANT or NCT. Additionally, DSG2 was significantly correlated with tumor size, PLNM, recurrence and vital status in 5 years, but not FIGO stage, pathologic type, differentiation grade, stromal invasion, LVSI, vaginal involvement and parametrial infiltration. High DSG2 expression predicted an unfavorable prognosis in early-stage CC. As PLNM was the most important risk factor for CC development, we investigated the relationship between DSG2 and PLNM by IHC, qRT-PCR and western blot analyses. All experiments showed that DSG2 was more highly expressed in the PLNM group than in the non-PLNM group. Moreover, high DSG2 expression was associated with high LMVD. Furthermore, our in vitro studies demonstrated that knockdown of DSG2 inhibited the CC cell proliferative capacity and migration ability. In conclusion, DSG2 was a novel tumor promoter in CC, and probably promoted cancer development by promoting the occurrence of PLNM.

However, some studies showed that the downregulation of DSG2 promoted the proliferation and metastasis of cancer cells because desmosome downregulation decreases adhesion junctions to drive tumor development and early invasion. The reasons why our results were contrary to some studies were probably as follows. First, as we found above, DSG2 played a different role in different kinds of cancer. Second, our results showed that high DSG2 expression was correlated with high LMVD, indicating that DSG2 may promote the occurrence of PLNM by promoting lymphangiogenesis. Cell adhesion was not an important factor in CC progression. Third, DSG2 may be a component of the metastasis pathway, regulating cell migration indirectly.

DSG2 plays a different role in different kinds of cancer. With TCGA data mining, we found that high DSG2 expression was correlated with the unfavorable prognosis of BLCA, brain LGG, LUAD, PAAD and UCEC, while high DSG2 expression was correlated with the favorable prognosis of COAD, KIRC and KIRP. These findings were consistent with those of previous reports. DSG2 is probably a novel biomarker of cancers but has different functions in different cancers.

Desmosomal cadherins are a component in cell–cell junctions, which are involved in the process of intercellular communication, signal transduction and cell proliferation [20]. In addition to regulating cell adhesion, DSG2 influenced cell proliferation and invasion by regulating the signaling pathway. It could be upstream or downstream of a pathway. The coexpression analyses results showed that cell division, positive regulation of transferase activity, positive regulation of cell migration and the EGFR upregulation pathway were significantly enriched among the positively correlated genes, revealing that DSG2 is involved in the process and metastasis of CC. These results were consistent with those of previous reports. Cai et al. [12] showed that knockdown of DSG2 suppressed non-small cell lung cancer cell proliferation by targeting p27 and CDK2. Kamekura et al. [10] reported that DSG2 and DSC2 played opposite roles in colon cancer cell proliferation. The loss of DSG2 suppressed cell proliferation through the altered phosphorylation of EGFR, Src and Erk protein. Overmiller et al. [21] suggested that in skin squamous cell carcinoma, DSG2 stimulated cell growth and migration by positively regulating EGFR levels and signaling through a c-Src and Cav1-dependent mechanism using lipid rafts as signal modulatory platforms. Brennan-Crispi et al. [22] showed that in skin basal cell carcinoma and squamous cell carcinoma, DSG2 enhanced canonical hedgehog signaling downstream of Ptc1 to promote cancer development through the activation of phosphorylated Stat3 and regulation of Gli1 expression. Katharina et al. [23] identified a novel promigratory pathway of pancreatic cancer cells in which the loss of DSG2 reduces the levels of plakoglobin via deregulated MAPK signaling. All of the above results showed that DSG2 was involved in various signaling pathways, such as the EGFR and MAPK signaling pathways as well as cell cycle pathways, indicating its important function in signaling pathway regulation.

Our study was the first to investigate the relationship between DSG2 expression and lymphangiogenesis. Coexpression analyses showed that DSG2 was positively correlated with CCBE1, which positively regulated lymphangiogenesis, while it was negatively correlated with VASH1, which negatively regulated lymphangiogenesis. An experiment was conducted to detect LMVD in tissue, which has not been reported in previous studies, and high LMVD was found to be associated with high DSG2 expression, indicating that DSG2 probably increased the lymphangiogenesis of cancer.

In conclusion, our current study was the first to show that DSG2 was overexpressed in CC tumorigenesis and that DSG2 knockdown repressed CC cell proliferation and migration. However, further mechanisms and signaling pathways underlying the role of DSG2 in CC remain to be defined.

## Conclusions

Based on the above data, we drew a conclusion that DSG2 was a biomarker that promotes CC cells proliferation and metastasis and is correlated with poor prognosis in early-stage CC. These findings facilitated us to discover novel targets for the therapy of patients with CC.

## Supplementary information

**Additional file 1. Supplemental Figure S1–S2 : Figure S1.** The bioinformatic analyses of prognosis-relative gene. **a** GO terms identified in the GO analysis for correlated coding genes in the cell component categories with 5 minimum *P-adjusted* values. GO terms identified in the GO analysis for correlated coding genes in the molecular function categories with 5 minimum *P *values (All *P-adjusted* value = 1). **b** Chromosome distribution of prognosis-relative gene. **c** Protein–protein interaction network of prognosis-relative gene. **d** GO terms identified in the GO biological process analysis for negatively coexpressed genes in categories with 20 minimum P-adjusted values. Oncogenic signature for negatively coexpressed genes with 20 minimum P-adjusted values. The number of enriched oncogenic signatures for negatively correlated genes was only 18. **Figure S2.** The effect of siRNA on CC cells detected by qRT-PCR (**a**) and Western blot (**b**). ****P < 0.0001.

## Data Availability

The datasets used and analyzed during the current study are available from the corresponding author on reasonable request.

## Abbreviations

CC: Cervical cancer
TCGA: The Cancer Genome Atlas
DSG2: Desmoglein-2
IHC: Immunohistochemistry
qRT-PCR: Quantitative real-time PCR
OS: Overall survival
CCK-8: Cell Counting Kit-8
MMP1: Matrix metallopeptidase 1
CA9: Carbonic anhydrase IX
HOXA1: Homeobox A1
SERPINB3: Serine protease inhibitor B3
ANT: Adjacent noncancerous tissue
PLNM: Pelvic lymph node metastasis
LVSI: Lymphovascular space invasion
NSCLC: Non-small cell lung cancer
KM: Kaplan–Meier
GO: Gene Ontology
KEGG: Kyoto Encyclopedia of Genes and Genomes
PPI: Protein–protein interaction
FDR: False discovery rate
DEA: Differential expression analyses
NCT: Normal cervical tissue
BLCA: Bladder urothelial carcinoma
LGG: Brain lower-grade glioma
LUAD: Lung adenocarcinoma
PAAD: Pancreatic adenocarcinoma
UCEC: Uterine corpus endometrial carcinoma
COAD: Colon adenocarcinoma
KIRC: Kidney renal clear cell carcinoma
KIRP: Kidney renal papillary cell carcinoma
LMVD: Lymphatic microvessel density
