# Supplementary material for: DSG2 expression is correlated with poor prognosis and promotes early-stage cervical cancer
Source: Cancer Cell Int. 2020 Jun 3;20:206. doi: 10.1186/s12935-020-01292-x (PMC7268232; doi:10.1186/s12935-020-01292-x)

**DSG2 expression is correlated with poor prognosis and promotes early-stage cervical cancer**

Shuhang Qin^1^, Yuandong Liao^1^, Qiqiao Du^1^, Wei Wang^1^, Jiaming Huang^1^, Pan Liu^1^, Chunliang Shang^2^, Tianyu Liu^1^, Meng Xia^1^, Shuzhong Yao^1*^

**Figure S1.** The bioinformatic analyses of prognosis-relative gene. **a** GO terms identified in the GO analysis for correlated coding genes in the cell component categories with 5 minimum *P-adjusted* values. GO terms identified in the GO analysis for correlated coding genes in the molecular function categories with 5 minimum *P*~~-adjusted~~ values (All *P-adjusted* value=1). **b** Chromosome distribution of prognosis-relative gene. **c** Protein–protein interaction network of prognosis-relative gene. **d** GO terms identified in the GO biological process analysis for negatively coexpressed genes in categories with 20 minimum *P-adjusted* values. Oncogenic signature for negatively coexpressed genes with 20 minimum *P-adjusted* values. The number of enriched oncogenic signatures for negatively correlated genes was only 18.


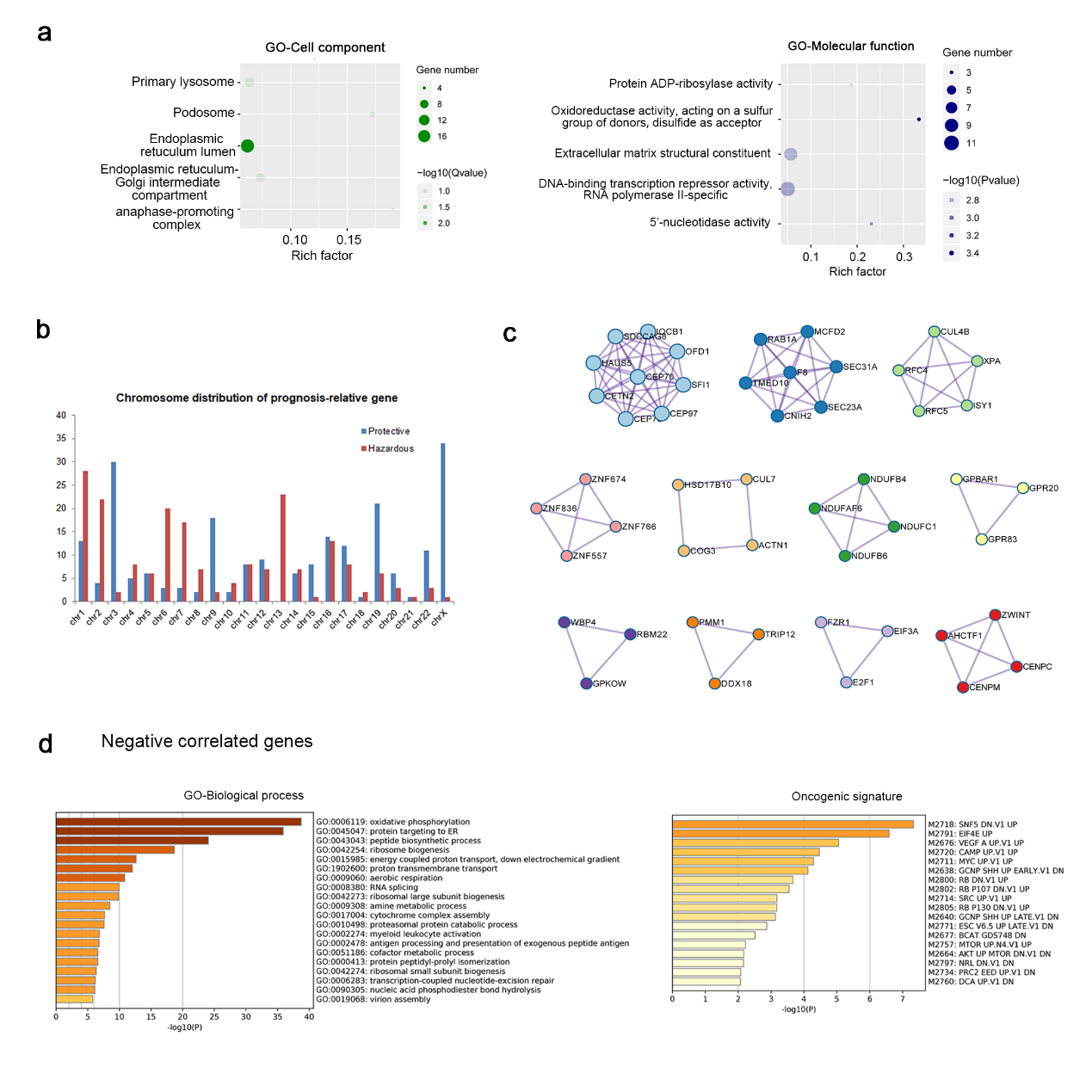


**Figure S2.** The effect of siRNA on CC cells detected by qRT-PCR (**a**) and Western blot (**b**). ****~~,~~ P < 0.0001.


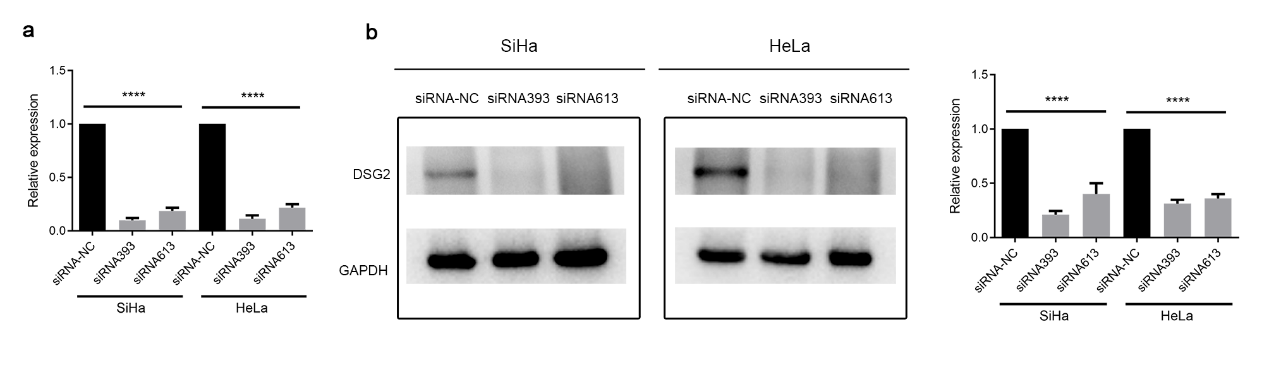

Supplement: Supplementary file 1 — Additional file 1. Supplemental Figure S1–S2 : Figure S1. The bioinformatic analyses of prognosis-relative gene. a GO terms identified in the GO analysis for correlated coding genes in the cell component categories with 5 minimum P-adjusted values. GO terms identified in the GO analysis for correlated coding genes in the molecular function categories with 5 minimum P values (All P-adjusted value = 1). b Chromosome distribution of prognosis-relative gene. c Protein–protein interaction network of prognosis-relative gene. d GO terms identified in the GO biological process analysis for negatively coexpressed genes in categories with 20 minimum P-adjusted values. Oncogenic signature for negatively coexpressed genes with 20 minimum P-adjusted values. The number of enriched oncogenic signatures for negatively correlated genes was only 18. Figure S2. The effect of siRNA on CC cells detected by qRT-PCR (a) and Western blot (b). ****P < 0.0001. [file 12935_2020_1292_MOESM1_ESM.docx]
